# Alterations in white matter fiber density associated with structural MRI and metabolic PET lesions following multimodal therapy in glioma patients

**DOI:** 10.3389/fonc.2022.998069

**Published:** 2022-11-14

**Authors:** Michel Friedrich, Ezequiel Farrher, Svenja Caspers, Philipp Lohmann, Christoph Lerche, Gabriele Stoffels, Christian P. Filss, Carolin Weiss Lucas, Maximilian I. Ruge, Karl-Josef Langen, Nadim J. Shah, Gereon R. Fink, Norbert Galldiks, Martin Kocher

**Affiliations:** ^1^ Institute of Neuroscience and Medicine (INM-1, -3, -4, -11), Research Center Juelich, Juelich, Germany; ^2^ Institute for Anatomy I, Medical Faculty and University Hospital Duesseldorf, Heinrich Heine University Duesseldorf, Duesseldorf, Germany; ^3^ Department of Stereotaxy and Functional Neurosurgery, Center for Neurosurgery, Faculty of Medicine and University Hospital Cologne, Cologne, Germany; ^4^ Department of Nuclear Medicine, University Hospital Aachen, Rheinisch-Westfaelische Technische Hochschule (RWTH) Aachen University, Aachen, Germany; ^5^ Center of Integrated Oncology (CIO), Universities of Aachen, Bonn, Cologne, and Duesseldorf, Cologne, Germany; ^6^ Department of General Neurosurgery, Faculty of Medicine and University Hospital Cologne, University of Cologne, Cologne, Germany; ^7^ Juelich-Aachen Research Alliance (JARA), Section JARA-Brain, Juelich, Germany; ^8^ Department of Neurology, University Hospital Aachen, Rheinisch-Westfaelische Technische Hochschule (RWTH) Aachen University, Aachen, Germany; ^9^ Department of Neurology, Faculty of Medicine and University Hospital Cologne, University of Cologne, Cologne, Germany

**Keywords:** glioma, multimodal therapy, PET/MR hybrid imaging, high-angular resolution diffusion-weighted imaging, white matter damage, tractography, fiber density imaging, constrained spherical deconvolution

## Abstract

**Background:**

In glioma patients, multimodality therapy and recurrent tumor can lead to structural brain tissue damage characterized by pathologic findings in MR and PET imaging. However, little is known about the impact of different types of damage on the fiber architecture of the affected white matter.

**Patients and methods:**

This study included 121 pretreated patients (median age, 52 years; ECOG performance score, 0 in 48%, 1-2 in 51%) with histomolecularly characterized glioma (WHO grade IV glioblastoma, n=81; WHO grade III anaplastic astrocytoma, n=28; WHO grade III anaplastic oligodendroglioma, n=12), who had a resection, radiotherapy, alkylating chemotherapy, or combinations thereof. After a median follow-up time of 14 months (range, 1-214 months), anatomic MR and O-(2-[^18^F]fluoroethyl)-L-tyrosine (FET) PET images were acquired on a 3T hybrid PET/MR scanner. Post-therapeutic findings comprised resection cavities, regions with contrast enhancement or increased FET uptake and T2/FLAIR hyperintensities. Local fiber density was determined from high angular-resolution diffusion-weighted imaging and advanced tractography methods. A cohort of 121 healthy subjects selected from the 1000BRAINS study matched for age, gender and education served as a control group.

**Results:**

Lesion types differed in both affected tissue volumes and relative fiber densities compared to control values (resection cavities: median volume 20.9 mL, fiber density 16% of controls; contrast-enhanced lesions: 7.9 mL, 43%; FET uptake areas: 30.3 mL, 49%; T2/FLAIR hyperintensities: 53.4 mL, 57%, p<0.001). In T2/FLAIR-hyperintense lesions caused by peritumoral edema due to recurrent glioma (n=27), relative fiber density was as low as in lesions associated with radiation-induced gliosis (n=13, 48% vs. 53%, p=0.17). In regions with pathologically increased FET uptake, local fiber density was inversely related (p=0.005) to the extent of uptake. Total fiber loss associated with contrast-enhanced lesions (p=0.006) and T2/FLAIR hyperintense lesions (p=0.013) had a significant impact on overall ECOG score.

**Conclusions:**

These results suggest that apart from resection cavities, reduction in local fiber density is greatest in contrast-enhancing recurrent tumors, but total fiber loss induced by edema or gliosis has an equal detrimental effect on the patients’ performance status due to the larger volume affected.

## Introduction

There is broad evidence that brain functions depend critically on the integrity of structural connections between cortical regions (1–6). These connections are built by axon bundles in the brain’s white matter and can be identified as single fibers or tracts composed of fiber groups using modern diffusion-weighted magnetic resonance imaging (DWI) techniques (7–9). Following multimodal treatment in glioma patients, fiber connections may become disrupted by structural tissue damage resulting from tumor resection, radiotherapy, alkylating chemotherapy, or combinations thereof (10–13), or by recurrent tumor growth (14). Apart from the fiber tracts originating in the primary, eloquent cortical regions, tissue damage may affect larger and wider distributed white matter areas (15–17) involving structural connections of multiple functional networks (18). Therefore, glioma patients often develop deficits in cognition, general performance (19), and quality of life that increase with the duration of survival and intensity of therapy (15, 20).

While the gross structural tissue changes induced by neurosurgical tumor resection, radiation, local tumor recurrence and edema can be readily made visible by standard magnetic resonance imaging (MRI) and amino acid positron emission tomography (PET) such as O-(2-[^18^F]fluoroethyl)-L-tyrosine (FET) PET, the resulting damage to white matter microstructural integrity remains to be elucidated (21, 22). In principle, fiber tractography methods, based on DWI aiming to identify individual interconnecting fibers at the submillimeter level, are best suited to answer this question. Diffusion tensor imaging (DTI), used to model the MR signal behavior in DWI, is based on a simple diffusion tensor model and is widely established in clinical practice, aiding to estimate white matter fiber orientation (23). However, the model is *a priori* unable to resolve multiple fiber orientations, which are present in approximately 90% of the voxels, causing missing or false positive fibers (23, 24). Advanced methods that can overcome the former limitations use DWI data acquired within the so-called high-angular resolution diffusion-weighted MR imaging (HARDI) framework. Amongst these methods, constrained spherical deconvolution (CSD) (25) has been shown to improve the assessment of complex, intra-voxel fiber configuration significantly. Thus, fiber-tracking procedures based on advanced DWI methods allow a more accurate estimation of complex fiber architectures (7) and are increasingly used for planning the extent of resection in brain tumors adjacent to eloquent areas (23, 26–32).

We hypothesize here that, apart from resection, structural brain damage due to radiation or tumor recurrence, as indicated by pathologic MRI and FET PET findings, has a differential impact on local fiber density and affects the patient’s overall performance status to varying degrees.

## Patients and methods

### Patient characteristics

The patient group consisted of 121 patients (73 males, 48 females; mean age, 51.6 ± 11.6 years) with histomolecularly characterized glioma (World Health Organization (WHO) grade IV glioblastoma, n=81; WHO grade III anaplastic astrocytoma, n=28; WHO grade III anaplastic oligodendroglioma, n=12) according to the WHO classification of 2016 (33), who underwent resection, radiotherapy, alkylating chemotherapy, or combinations thereof (**Table 1**). Most of the patients (77, 64%) received their primary treatment between 2016 and 2019 in one of the 4 university hospitals of the comprehensive cancer center ‘Center for Integrative Oncology Aachen-Bonn-Cologne-Duesseldorf’, and another 21 (17%) were treated at another university hospital (Frankfurt). Complete resection as determined from early postoperative contrast-enhanced MR was achieved in 88 patients (73%), while the others had partial resection or stereotactic biopsy only. At time of imaging, adjuvant radiotherapy (60 Gy in most cases) had been applied in 112 (93%) and simultaneous and/or adjuvant chemotherapy comprising temozolomide, temozolomide and lomustine (CCNU) or procarbacine/CCNU/vincristine (PCV) in 108 (89%). Where ever possible, the final diagnosis was based on the presence of a IDH (isocitrate-dehydrogenase) mutation and the 1p-19q loss-of-heterozygosity status. Of note, therapy was initiated between 2000 and 2015 in some patients, so molecular characteristics were not available. Between 2018 and 2020, structural MRI and metabolic PET findings after treatment were evaluated in all patients using anatomical MRI and FET PET acquired on a 3T hybrid PET/MR scanner (Siemens Trim-TRIO/BrainPET, Siemens Medical Systems, Erlangen). The median interval between treatment initiation and imaging was 14 months (range, 1-214 months). Of note, 14 patients were examined more than 60 months (5 years) after therapy initiation. Regarding general performance status, 58 patients (48%) had an ECOG score of 0 (fully active, able to carry on all pre-disease performance without restriction), 56 (46%) were grade 1 (restricted in physically strenuous activity, but ambulatory and able to carry out work of a light or sedentary nature), and 6 (5%) were grade 2 (ambulatory, capable of all self-care, up and about more than 50% of waking hours, but unable to work) (19). All patients were free from major depression and seizures. A total of 81 patients (67%) had mild neurological (48%) or other symptoms (fatigue, vertigo, 19%) without requiring assistance for personal needs.

**Table 1:** Patient characteristics.

	n	%
**Gender** (male/ female)	73/ 48	60/ 40
**ECOG score** (0/ 1/ 2/ 3)	58/ 56/ 6/ 1	48/ 46/ 5/ 1
**Tumor type**		
GBM: IDH-wt/ IDH-mut/ NOS	67/ 10/ 4	56/ 8/ 3
AA: IDH-wt/ IDH-mut/ NOS	5/ 16/ 7	4/ 13/ 6
AOD: IDH-mut-1p-19q-codel	12	10
Glioma Grade 3/ Grade 4	40/ 81	33/ 67
IDH-wt or NOS/ IDH-mut	88/ 37	69/ 31
**Tumor location**		
Left frontal/ parietal/ temporal/ occipital	30/ 8/ 22/ 5	25/ 7/ 18/ 4
Right frontal/ parietal/ temporal/ occipital	28/ 8/ 16/ 4	23/ 7/ 13/ 3
**Primary treatment^#^**		
Biopsy/ partial/ complete resection	19/ 14/ 88	16/ 11/ 73
Radiotherapy yes/ no	112/ 9	93/ 7
Temozolomide	76	63
Temozolomide + CCNU	27	22
PC/ PCV	5	4
**Number of treatment interventions^#^**		
Surgery* (1/ 2/ 3/ 4)	101/ 17/ 2/ 1	83/ 14/ 2/ 1
Radiotherapy series (0/ 1/ 2)	7/ 100/ 14	6/ 83/ 12
Chemotherapy courses (0/ 1/ 2/ 3)	10/ 91/ 16/ 4	8/ 76/ 13/ 3
**Neurological symptoms**		
None	40	33
Paresis	29	24
Aphasia	17	14
Visual field/ diplopia	12	10
Other symptoms	23	19
	**mean ± SD**	**median (range)**
**Age (years)**	51.6 ± 11.6	51.9 (28.1 - 73.8)
**Radiation dose (Gy)**	59.3 ± 2.6	60.0 (40.1 - 62.0)
of first radiation series (n=114)		
**Interval (months)**	30.4 ± 43.0	14.4 (0.6 - 213.7)
between therapy and imaging		

ECOG, Eastern Cooperative Oncology Group; GBM, glioblastoma multiforme; AA, anaplasticastrocytoma; AOD, anaplastic oligodendroglioma; IDH-wt/-mut, mutation status in the isocitrate dehydrogenase gene (wildtype/mutant); 1p-19q-codel, 1p/19q-codeletion; NOS, not otherwise specified; CCNU, lomustine; PCV, procarbacine/CCNU/vincristine; ^#^received prior to imaging; *including biopsy and resection.

A control group of 121 healthy subjects was obtained from the 1000BRAINS cohort study (34) that investigates environmental and genetic influences on inter-individual variability in brain structure, function, and connectivity in the aging brain. Controls were matched for gender (males, n=75; females, n=46), age (mean 51.7 ± 11.5 years), and educational status using propensity score matching (35). Both cohorts have been analyzed in an earlier study presented by our group (36).

### Hybrid PET/MR imaging

In all patients, FET PET, as well as anatomical and diffusion-weighted MR images, were obtained from the 3T hybrid PET/MR scanner equipped with a birdcage-like quadrature transmitter head coil mounted on the couch, an 8-channel receiver coil and a PET insert consisting of 72 rings (axial field-of-view, 19.2 cm; center spatial resolution, 3 mm FWHM). The PET image data were corrected for random and scatter coincidences as well as for dead time, attenuation (based on a T1-weighted anatomical MRI scan), and motion before OPOSEM (Ordered Poisson Ordinary Subset Expectation Maximization) reconstruction (2 subsets, 32 iterations), with software provided by the manufacturer (37).

The MRI protocol comprised a 3D high-resolution T1-weighted magnetization prepared rapid acquisition gradient echo (MPRAGE) native scan (176 slices; TR=2250 ms; TE=3.03 ms; FoV=256×256 mm^2^; flip angle=9°; voxel size=1×1×1 mm^3^), a contrast-enhanced MPRAGE scan recorded after injection of gadolinium-based contrast agent, a T2-weighted sampling perfection with application optimized contrasts (SPACE) scan (176 slices; TR=3.2 ms; TE=417 ms; FoV=256×256 mm^2^; voxel size=1×1×1 mm^3^), and a T2-weighted fluid-attenuated inversion recovery (T2/FLAIR) scan (25 slices; TR=9000 ms; TE=3.86 ms; FoV=220×220 mm^2^; flip angle=150°; voxel size=0.9×0.9×4 mm^3^).

The HARDI measurements were performed with a double-echo diffusion-weighted echo-planar imaging (EPI) sequence. The protocol parameters were: 55 slices, TR=8 s; TE=112 ms; *b*-values (gradient directions)=0 (13, interleaved) and 2700 s/mm^2^ (120); voxel size=2.4×2.4×2.4 mm^3^). An additional non-diffusion-weighted volume was acquired with the same settings but a reverse phase-encoding direction for the purpose of EPI distortion correction. The healthy subjects were measured on a stand-alone MRI scanner (3T Siemens Tim-TRIO), identical to the MR component of the hybrid PET/MR system. The body coil was used for transmission and a 32-channel receive-only head coil for signal reception. Before the *in vivo* measurements, a phantom study was performed as a control that confirmed an equal signal level, quality and signal-to-noise ratio values between both scanners.

### Lesion segmentation

The local fiber density was evaluated in four different types of imaging findings: i) hypointense resection cavities, ii) contrast-enhancing lesions, iii) T2/FLAIR hyperintense regions, and iv) lesions with pathologically increased FET uptake defined by a tumor-to-brain ratio (TBR) >1.6. A fully automated software based on deep-learning algorithms (HD_GLIO-AUTO) was used to segment T2/FLAIR hyperintense regions and contrast-enhancing lesions (38).

Resection cavities were manually contoured using the medical 3D segmentation software ITK-SNAP (http://www.itksnap.org, vs. 3.8.0, Universities of Pennsylvania and Utah, USA). The resection cavities were mostly filled with cerebro-spinal fluid but sometimes also comprised complex, intermingled structures of undeterminable origin. The mask of areas with increased FET uptake originated from a semi-automatic segmentation that identified all voxels with a TBR above 1.6, which was histologically validated and is highly predictive for glioma tissue (39). Finally, all masks were visually examined and manually corrected by i) removing spurious small-segmented regions that were not connected to the primary lesion, and ii) padding of necrotic areas surrounded by contrast-enhancing or FET-enhancing tissue.

T2/FLAIR lesions can be caused by both peritumoral edema and radiation-induced gliosis which may be present simultaneously and difficult to distinguish. Therefore, the prevailing characteristics were used to assign a single classification to a selection of patients. Such, T2/FLAIR lesions were classified as perifocal edema in n=27 patients with recurrent tumors >10 mL in both the contrast-enhancing and FET images. In opposite, T2/FLAIR hyperintensities were classified as radiation-induced gliosis in n=13 patients in the almost complete absence of contrast enhancement and FET uptake (<0.1 mL) and a time interval >6 months from local irradiation.

### Tractography and local fiber density

Advanced DWI and tractography methods have rarely been used to characterize or quantify brain tissue damage caused by infiltrative tumor growth or treatment effects other than surgery (40). This reluctance might be related to the observation that most fiber tracking methods in brain regions affected by recurrent tumor or other structural changes yield inconsistent or biased results, usually leading to a severe underestimation of fiber density (28, 32, 41, 42). Therefore, we applied a recently developed modification of a widely used fiber-tracking method that allows for reasonable identification of the fibers passing through and near tumorous tissue and the surrounding brain structures (43). In short, the applied constrained spherical deconvolution (CSD) method (25) assumes that the diffusion-weighted MRI signal results from the spherical convolution of a response function with the underlying fiber orientation distribution function (FOD). The response functions are tissue-type specific and describe the expected MR signal of a pure white matter (single oriented white matter fiber bundle), gray matter, or cerebrospinal fluid image voxel. The estimated white matter FODs in the original, single-shell CSD model (25) are usually distorted by signal contributions from different tissue types within the voxels. This problem has been addressed by the advanced multi-shell multi-tissue CSD (MSMT-CSD) method, which also considers the signal contributions of gray matter and cerebrospinal fluid and exploits their different response properties at different *b*-values (44). However, it has also been shown that MSMT-CSD underestimates or excludes white matter FODs in tumor tissue, since such areas are often misclassified as gray matter-like tissue (43). In contrast, the novel single-shell 3-tissue CSD (SS3T-CSD) method considers different tissue types from single-shell (single *b*-value plus non-diffusion weighted images) HARDI data and estimates white matter FODs as bias-free as possible, even within different compartments of a tumor (43, 45, 46). The method is implemented in the toolkit MRtrix3Tissue (https://3tissue.github.io, accessed on 1.3.2021), a fork of the widely used fiber tracking toolkit MRtrix3 [https://www.mrtrix.org, accessed on 1.3.2020 (47)].

The MRtrix3Tissue toolkit steps were embedded in the following processing pipeline. The image corrections were passed from MRtrix to the FSL toolbox [FSL version 5.0, https://fsl.fmrib.ox.ac.uk/fsl (48)] and the ANTs software suite (https://github.com/ANTsX/ANTs, accessed on 3.1.2020). First, the HARDI data were subjected to EPI distortion correction using the script “topup”. Second, eddy-current and motion distortion correction were performed using the FSL tool “eddy”, both scripts available in FSL. Afterwards, a bias field correction based on the N4ITK algorithm was executed by the ANTs software suite. The white matter, gray matter, and cerebrospinal fluid response functions were estimated from the preprocessed HARDI data using an unsupervised method (46). Afterwards, SS3T-CSD was performed to obtain white matter-, gray matter- and cerebrospinal fluid-like FODs in all voxels (45). The response functions for each tissue compartment were averaged across all patients and subjects in order to ensure that the FODs were comparable within the group study. In addition, the FODs were subjected to a global intensity normalization (49).

**Figure 1:**
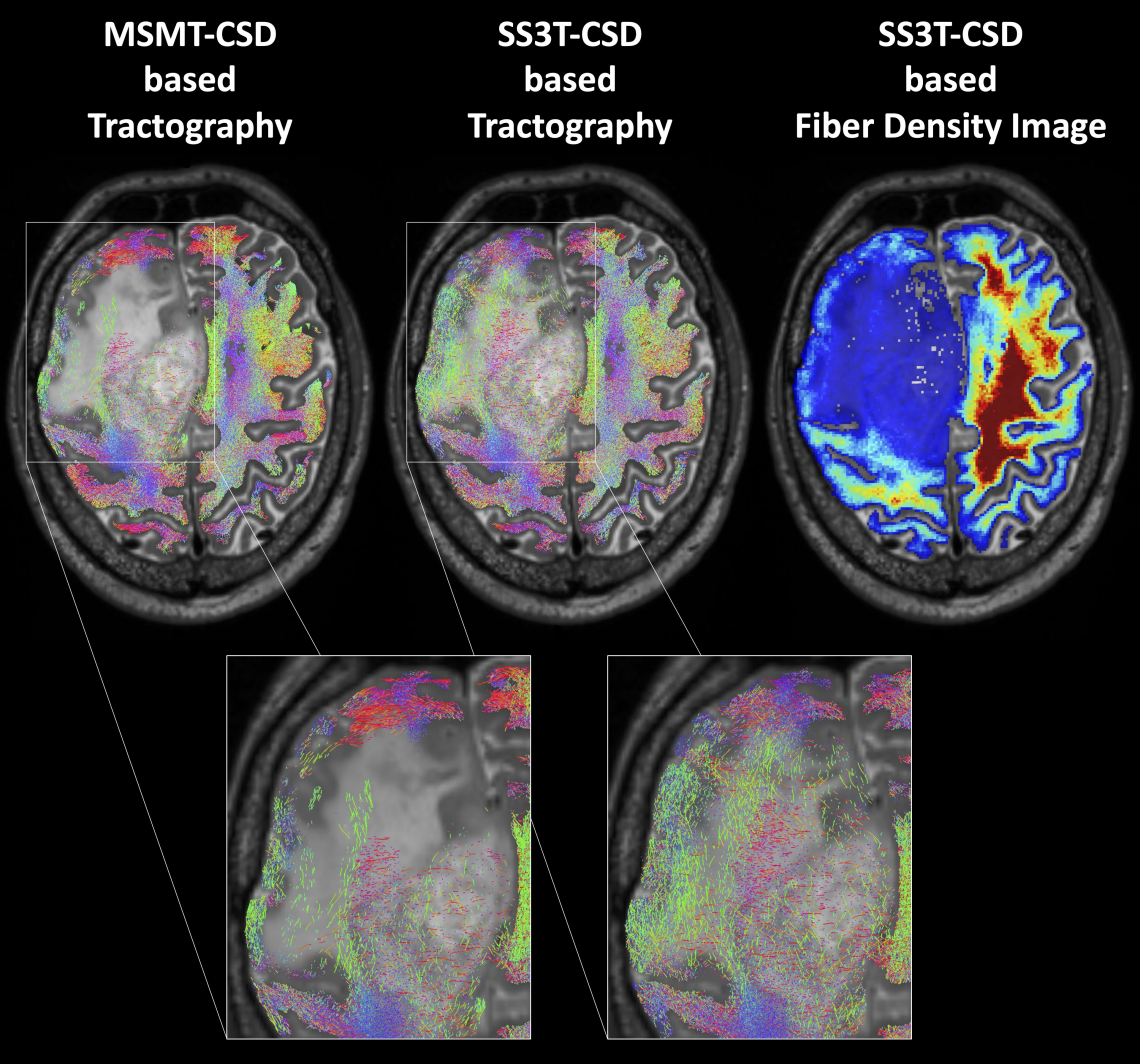
Probabilistic whole-brain tractography and fiber density image in a patient with recurrent glioma and perifocal edema. In normal brain tissue, a method that increases the biological plausibility is applied (Anatomically-Constrained Tractography), while this condition is relaxed in pathologically altered brain regions. In contrast to the standard multi-shell multi-tissue constrained spherical deconvolution (CSD) based tractography of MRtrix3 (MSMT), the advanced MRtrix3tissue method using the single-shell 3-tissue CSD algorithm (SS3T) detected an adequate number of fibers also within the tumorous or edematous tissue.

**Figure 2:**
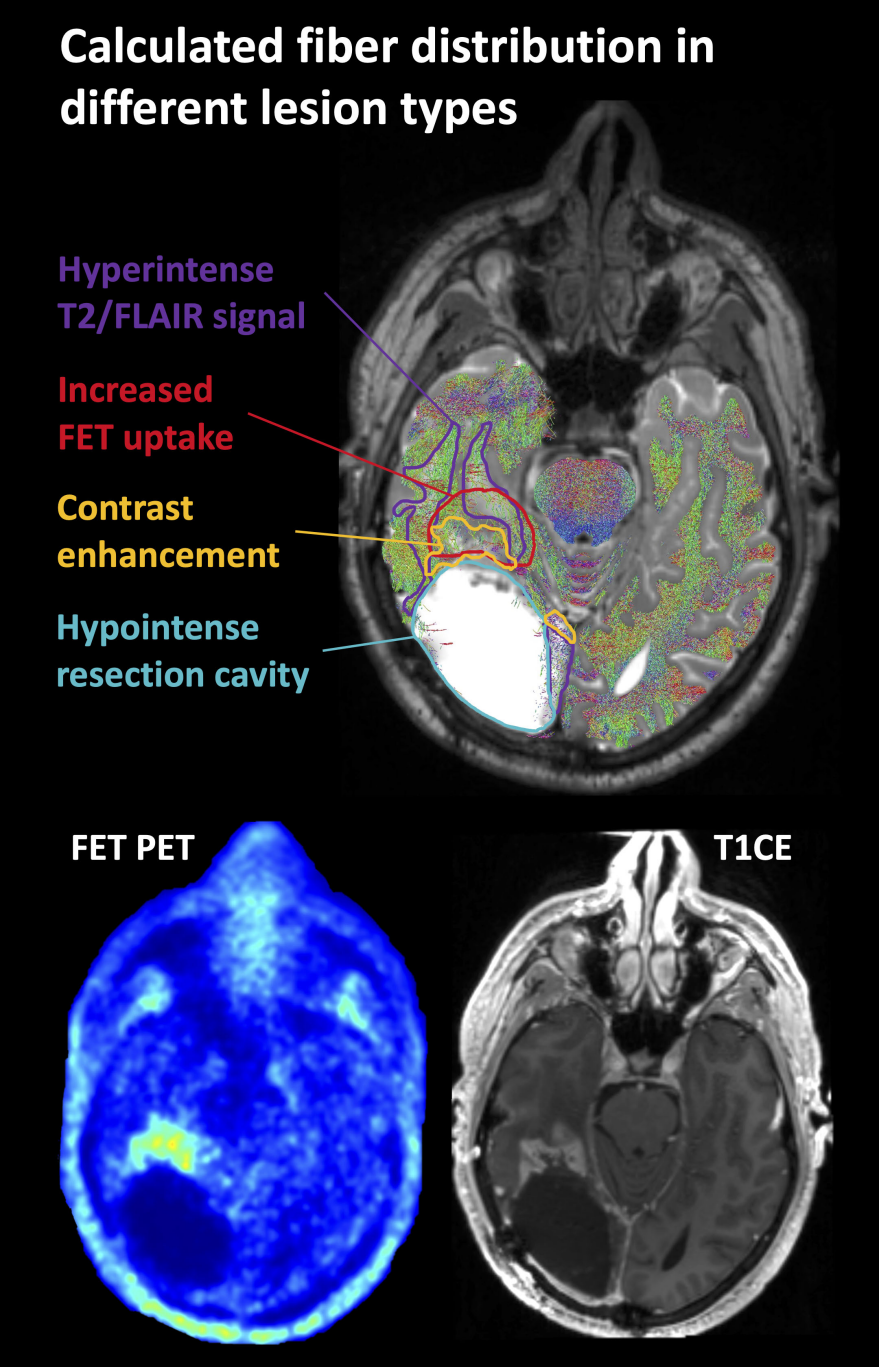
Representative case of lesion segmentation superimposed on a tractography image in a patient who had undergone surgery and radiochemotherapy and developed local recurrence near the resection cavity. FET, O-(2-[^18^F]fluoroethyl)-L-tyrosine; T2/FLAIR, T2-weighted fluid-attenuated inversion recovery; T1CE, T1-weighted contrast-enhancing image.

Finally, in order to increase the biological plausibility of the fiber tractograms, the method called Anatomically-Constrained Tractography (available as part of MRtrix) which makes several assumptions about the behavior of healthy neuronal fibers in terms of their propagation and termination was applied to the obtained fiber tractograms (50). These assumptions were relaxed in all areas of segmented pathological tissue using a compound lesion mask containing all segmented lesion types. Apart from the default settings, the option “backtrack” was activated, the number of seed points was fixed at 4 million and restricted to a brain mask, and the cutoff value for the FOD amplitude was set to 0.01. Lastly, the tractography data were converted into fiber density images with an isotropic voxel size of 1 mm^3^. All image processing steps were also performed for the control group, except for the EPI distortion correction due to the lack of the corresponding sequence with reversed phase-encoding. In **Figures 1**, **2**, representative results for the applied tractography methods and lesion segmentation are depicted. Although the lack of the EPI distortion correction could theoretically have led to an inward-facing deformation mainly of the frontal tracts in the healthy subjects, there was no indication that this happened in the fiber density images, probably due to the successful application of the spatial normalization to the MNI template (see next section).

### Effect of lesion type on local fiber density

The tractography methods supplied by MRtrix support the determination of the voxel-wise fiber density [also termed track density by the developers (51, 52)], which we here used to measure the integrity of local structural connectivity. For this purpose, all structural and fiber density images of the patients and healthy subjects as well as all lesion masks of the patients were registered from the individual patient space to the standard MNI space by the unified segmentation method of the SPM12 toolbox (Statistical Parametric Mapping Toolbox, https://www.fil.ion.ucl.ac.uk/spm/software/spm12/, Matlab R2017b, MathWorks, Natick, MA, USA). This method combines tissue segmentation with elastic registration (53). All further analyses were done in the MNI standard space. A map of the average fiber density in the control group (n=121) was computed from the individual fiber density images of the healthy subjects. Then, the mean fiber density within the lesion segments of the patients was computed and compared to the mean fiber density in the corresponding region of the average normal fiber density map. In order to check the validity of the fiber density estimation, it was also evaluated for the manually segmented resection cavities and later used for calculation of the total fiber loss and its impact on the ECOG performance status (see next section).

In order to analyze a possible correlation between FET PET uptake and local fiber density, TBR values were divided into 4 bins, starting from the histologically validated cut-off value for glioma tissue of 1.6 (binning thresholds, 1.6-2.6; 2.6-3.6; 3.6-4.6; >4.6). As the tumors also extended into fiber-free areas (gray matter and ventricles), the following measures were undertaken to make the resulting TBR-binned lesion segments comparable. Only patients with pathologically increased FET uptake located in a region with a reasonable homogenous underlying fiber density in the reference fiber density image (control group) were included. Thus, only patients for whom the standard deviation of the fiber density in the reference region was smaller than the mean fiber density itself were considered. Besides, tiny TBR-binned segments (i.e., <0.25 mL) were excluded within the patients, and one patient was discarded for whom the displaced fibers probably caused the fiber density to exceed the reference value. In the remaining 43 patients, the mean TBR within the TBR-binned segments was computed, and the mean relative fiber density was expressed as the ratio to the reference region.

### Impact of reduced fiber density on the ECOG performance status

The effect of reduced fiber density on performance status was examined to evaluate the clinical impact of reduced structural connectivity induced by different types of lesions. The total fiber loss caused by each lesion type was calculated from the relative reduction in fiber density [1 – (patient fiber density/reference fiber density)] of each segment multiplied by the corresponding segment volume. In patients where one or more lesion types were not present, the respective volumes were set to zero. The patient’s performance status was classified as either normal/unaffected (i.e., ECOG score of 0) or impaired (i.e., ECOG≥1).

### Statistical analysis

Statistical analysis was performed using the SPSS statistical software package (version 27, IBM Corporation, Armonk, New York, USA). All fiber density values were also converted to fractions relative to the reference values in the corresponding regions of the healthy subjects. To compare the absolute and relative fiber densities between patients and healthy subjects, the Mann-Whitney U test and one-sample Wilcoxon signed-rank tests (2-sided) were applied. For 3 lesion types (FET PET, T1CE and resection cavity), 1-2 outliers were each excluded from analysis. For evaluation of the differential effect of lesion types in the patients, the Kruskal-Wallis test (2-sided) and a post-hoc comparison by the Mann-Whitney U-test (2-sided) were applied. The relationship between FET uptake and relative fiber density was determined using linear regression analysis and a mixed linear model using the TBR as fixed effect and allowing for random variation of the constant term in each individual patient. The influence of the total fiber loss caused by different lesion types on performance status was examined using univariate and multivariate logistic regression analysis including a set of clinical variables as potential confounders. In all analyses, a p-value <0.05 was considered statistically significant.

## Results

**Figure 3:**
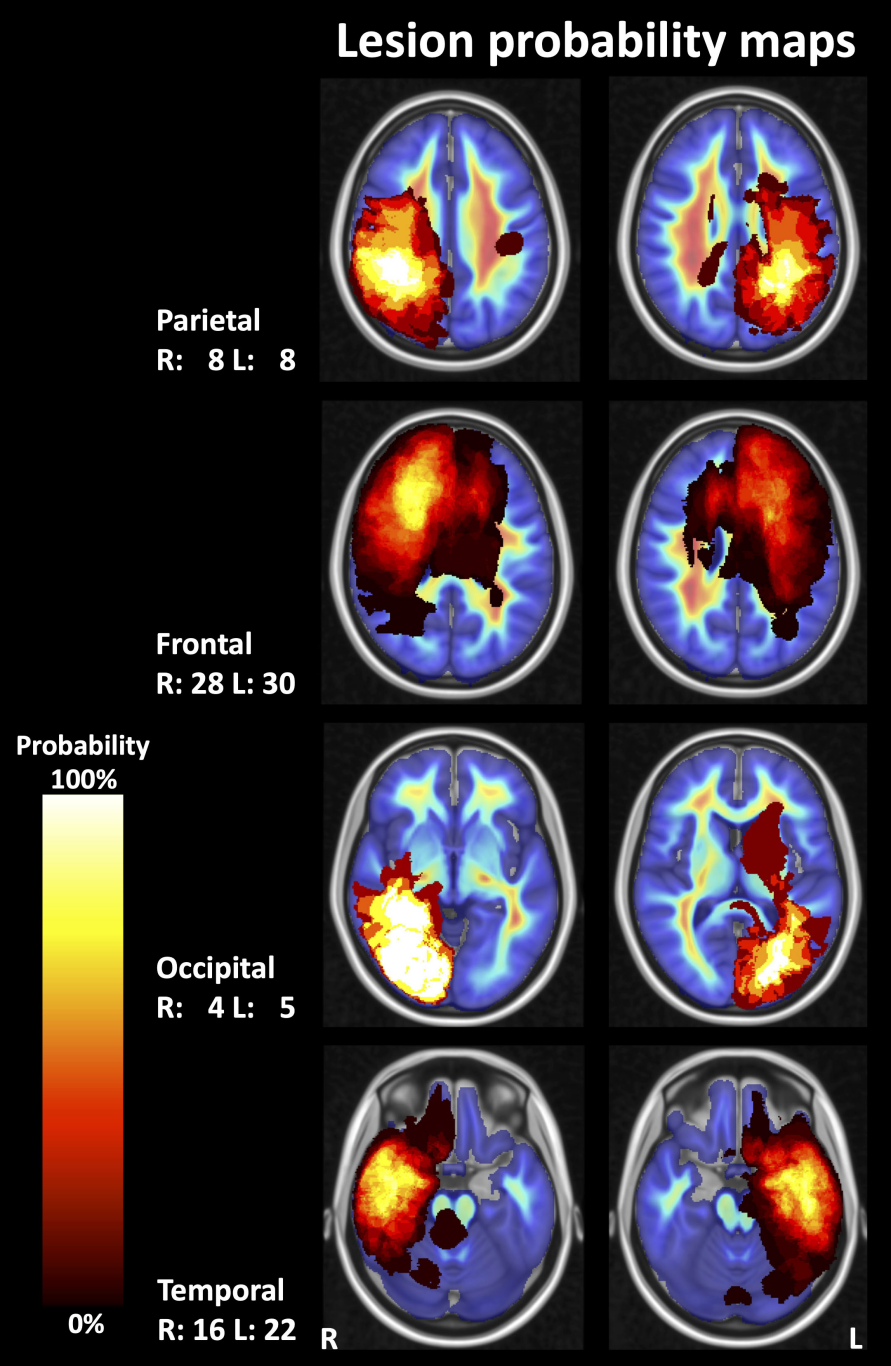
Probability maps for lesion localization. Compound lesion maps comprising T2-weighted fluid-attenuated inversion recovery (FLAIR) hyperintense lesions, contrast-enhancing lesions, resection cavities, and lesions with pathologically increased FET (O-(2-[^18^F]fluoroethyl)-L-tyrosine) uptake on PET images are shown superimposed on images depicting the mean fiber density in a control group (representative sections of the MNI-152 standard brain template). R, right; L, left; numbers, number of patients.

**Figure 4:**
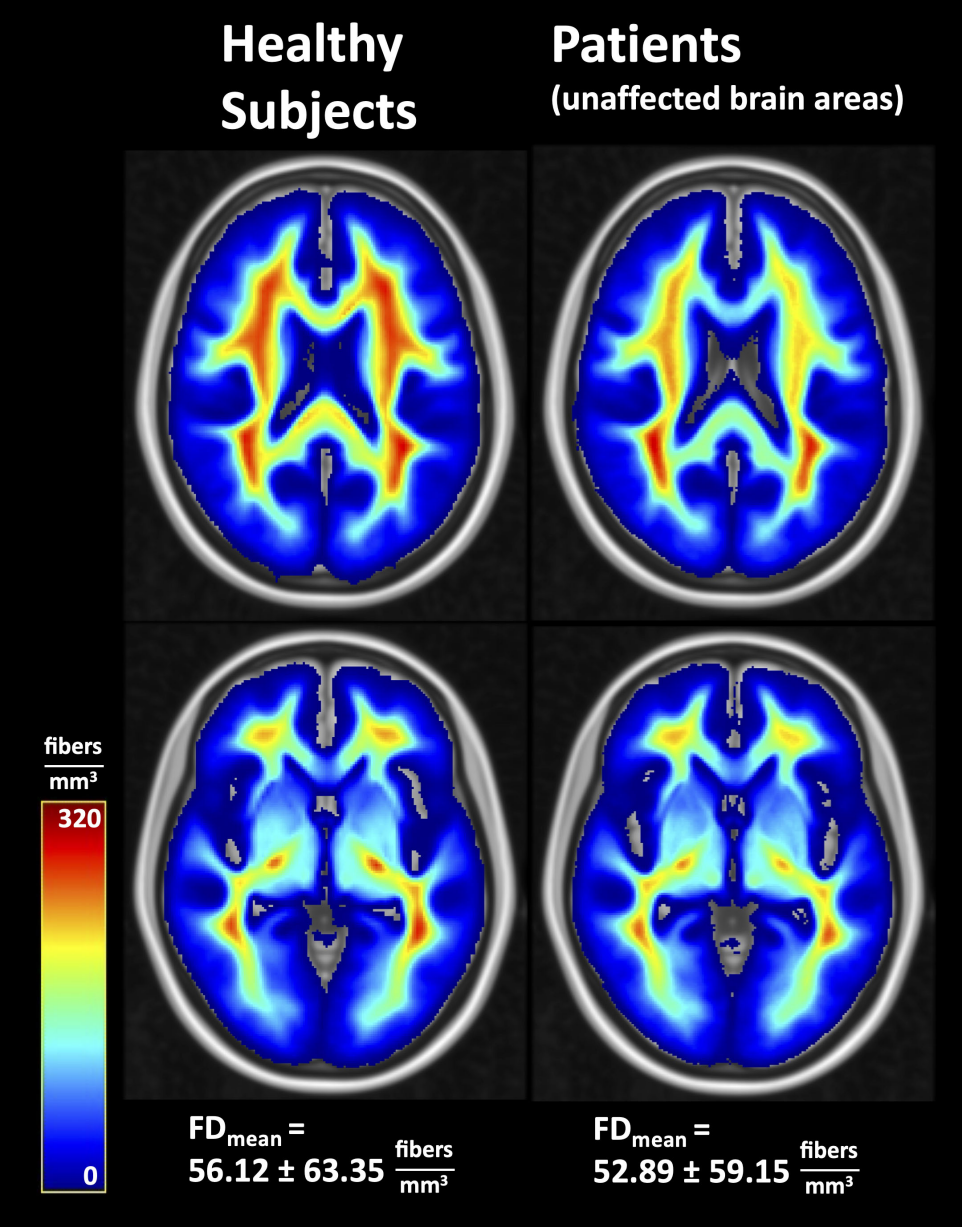
Average fiber densities in healthy subjects and in unaffected brain regions of the patients. FD, fiber density; FD_mean_, whole-brain mean fiber density.

The probability of lesion location is shown in **Figure 3**. Most lesions were located in the frontal and temporal lobes. Average fiber densities in the healthy subjects and in the unaffected brain regions of the patients are illustrated in **Figure 4**, indicating that the overall pattern of fiber tracts outside the lesions was maintained in the patients. However, as expected, some of the main tracts showed a reduced fiber density which we did not further evaluate here. As shown in **Table 2**, the median volume of resection cavities, contrast-enhancing regions, regions with increased FET uptake, and T2/FLAIR hyperintense regions amounted to 20.9, 7.9, 30.3, and 53.4 mL, respectively. A significant decrease in absolute fiber density was observed in all four major lesion types (p<0.001 in all cases).

**Table 2:** Volume and fiber density in lesions of different type.

Lesion type	n^#^	Lesion size (mL)mean ± SD (median)	Fiber density Healthy(fibers/mm^3^)	Patients (fibers/mm^3^)	Patients(% of reference)
Resection cavity	90	35.8 ± 40.3 (20.9)	45.1 ± 23.0	5.9 ± 7.3***	15.5 ± 21.1***
T1CE	99	17.2 ± 23.3 (7.9)	76.3 ± 43.5	26.9 ± 19.9***	42.9 ± 31.7***
FET PET (TBR>1.6)	79	39.5 ± 35.2 (30.3)	62.3 ± 30.6	27.8 ± 17.5***	49.3 ± 26.1***
T2/FLAIR	121	70.1 ± 55.5 (53.4)	107.6 ± 39.6	60.9 ± 29.1***	56.9 ± 16.3***
T2/FLAIR (edema)	27	121.1 ± 58.0 (131.8)	88.3 ± 23.2	40.7 ± 12.8***	48.1 ± 15.5***
T2/FLAIR (gliosis)	13	52.3 ± 45.7 (33.3)	120.4 ± 41.3	62.6 ± 24.8***	52.7 ± 12.5**

T1CE, T1-weighted contrast-enhancing; FET, O-(2-[^18^F]fluoroethyl)-L-tyrosine; FLAIR, fluidattenuated inversion recovery; TBR, tumor-to-brain ratio; ^#^patients affected; **p<0.01, ***p<0.001, Mann-Whitney U test (fibers/mm^3^), one-sample Wilcoxon signed-rank test (% of reference).

**Figure 5:**
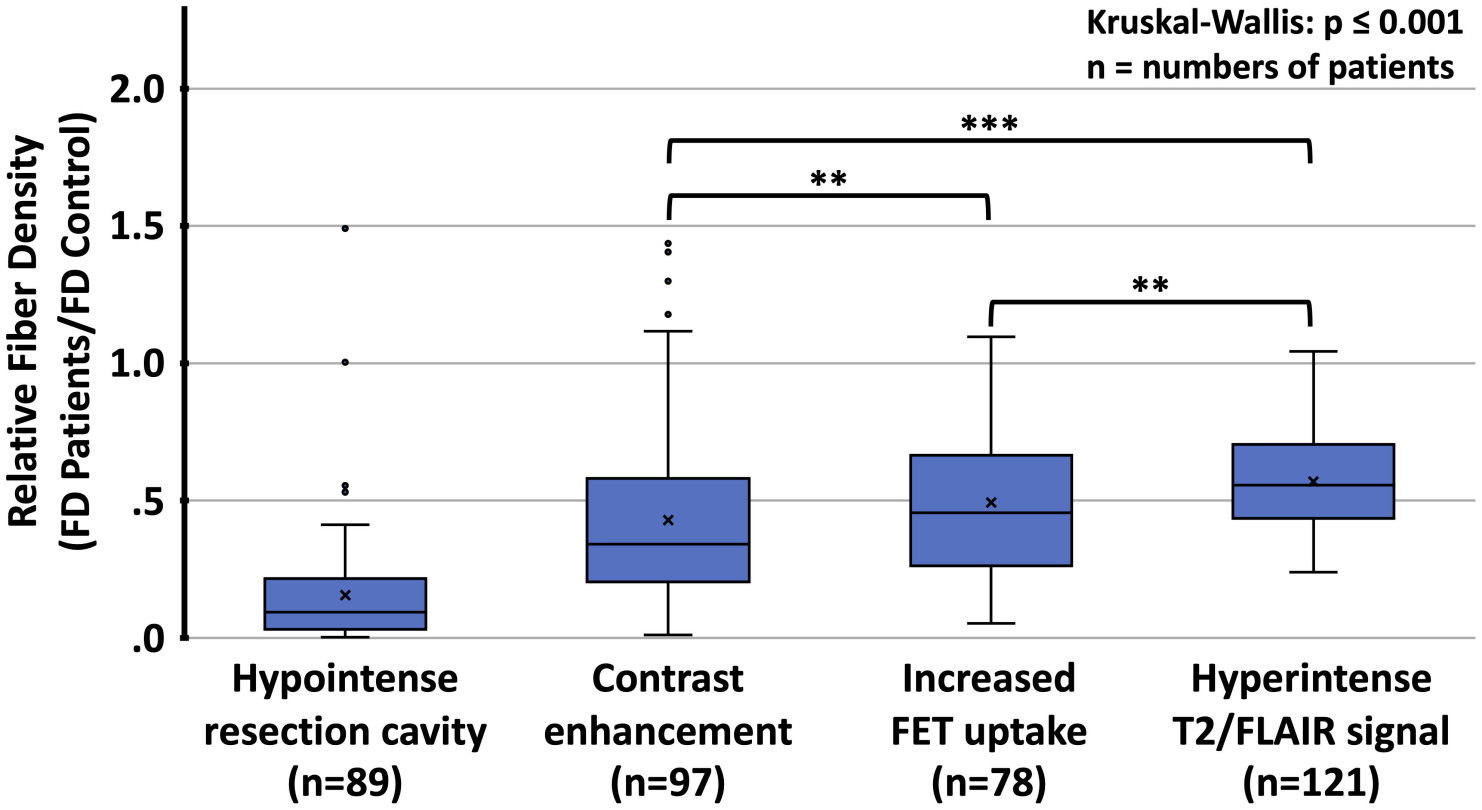
Distribution of relative fiber densities (fraction of fiber density (FD) of the corresponding region in healthy controls) for the respective imaging findings. FET, O-(2-[^18^F]fluoroethyl)-L-tyrosine; T2/FLAIR, T2-weighted fluid-attenuated inversion recovery (FLAIR) hyperintense lesions; **p<0.01, ***p<0.001, Mann-Whitney U test for comparison between different types of lesions.

The relative fiber densities (fraction of fiber density compared to the corresponding region in the healthy subjects) in different lesion types are shown in **Table 2** and **Figure 5**. The relative fiber density was most decreased in the resection cavities (resulting mean density 16%, p<0.001), followed by T1-weighted contrast-enhancing lesions (43%, p<0.001), lesions with pathologically increased FET uptake (49%, p<0.001) and T2/FLAIR hyperintense regions (57%, p<0.001) and depended significantly on lesion type (Kruskal-Wallis-test, p<0.001). A post-hoc analysis revealed that the contrast-enhancing lesions and FET uptake regions were associated with a significantly larger decrease in relative fiber density than the T2/FLAIR lesions (both p<0.001) and that the relative fiber density in contrast enhancing regions was significantly lower than in regions with pathologic FET uptake (p<0.01). Also, the relative fiber densities found in T1-enhancing lesions and in lesions with pathologic FET uptake did not differ significantly between grade 3 and grade 4 gliomas.

**Figure 6:**
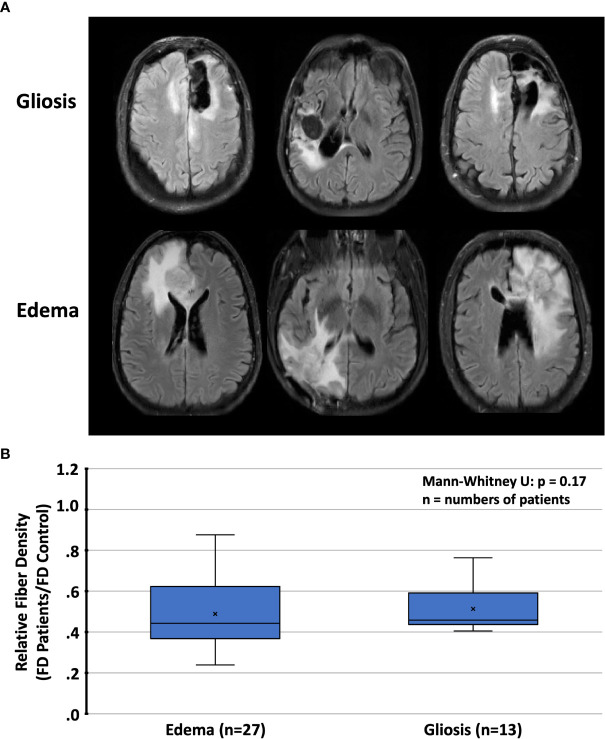
**(A)** Representative cases with T2/FLAIR hyperintense regions and **(B)** relative fiber densities (FD) in patients with radiation-induced gliosis or peritumoral edema. T2/FLAIR, T2-weighted fluid-attenuated inversion recovery.

**Figure 7:**
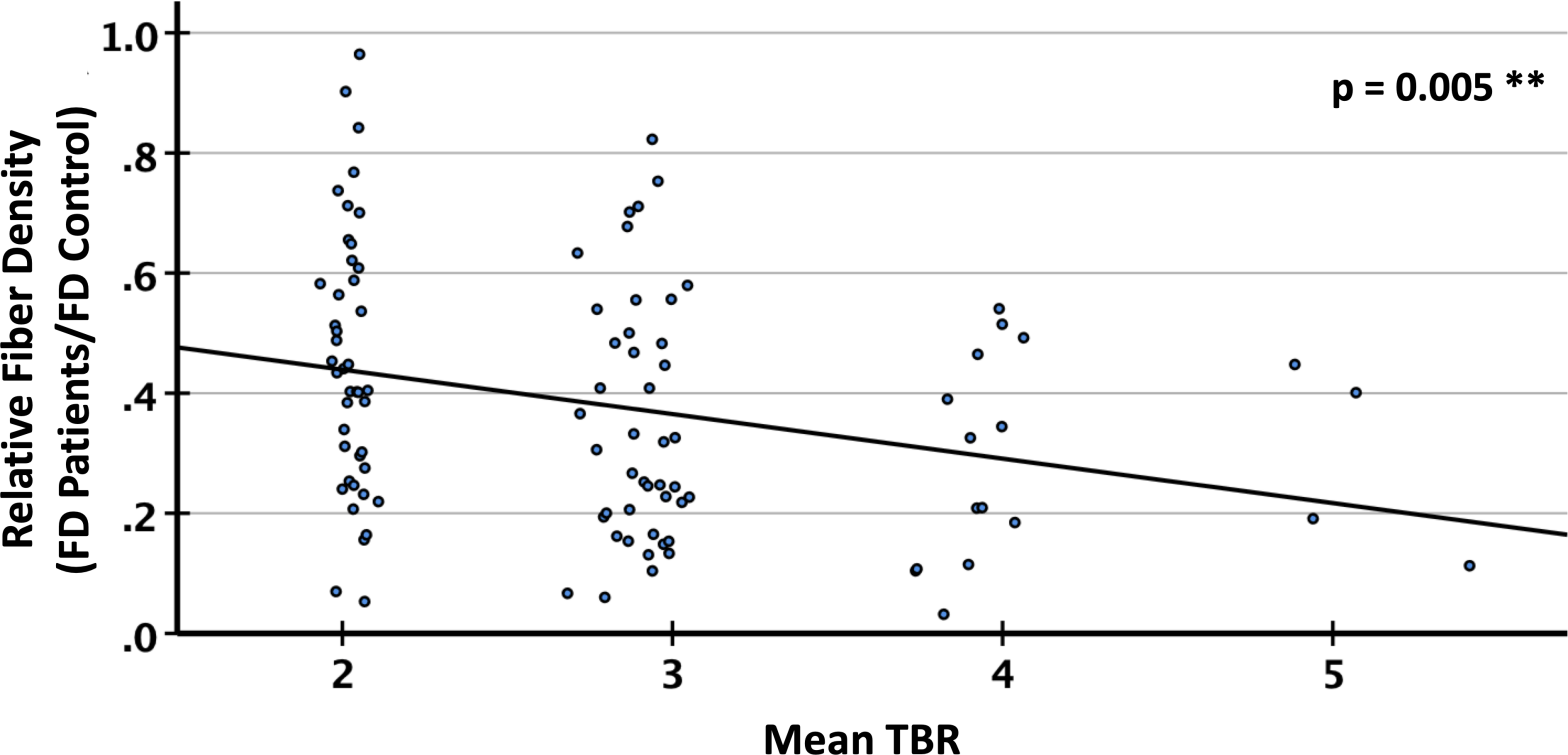
Relative fiber density (FD) in PET lesion segments with different tumor-to-brain ratio (TBR) of FET (O-(2-[^18^F]fluoroethyl)-L-tyrosine) uptake. Linear regression, R^2^=0.076, **p<0.01.

T2/FLAIR lesions, predominantly related to radiation-induced gliosis, were identified in n=13 patients (**Table 2**). Within these lesions, the mean relative fiber density amounted to 53% which was not significantly different from that measured in lesions dominated by tumor-related edema (n=27, 48%, p=0.17). Representative cases are presented in **Figure 6**. With regard to the FET uptake versus fiber density, a significant (p=0.005, R^2^= 0.076) inverse linear dependence (constant term 0.59, slope -0.074) of fiber density on the level of FET uptake (tumor-to-brain ratio, **Figure 7**) was observed. The mixed linear model confirmed the highly significant dependency of fiber density on TBR (p<0.001) and the inverse relationship (mean constant term 0.61, slope -0.084).

**Figure 8:**
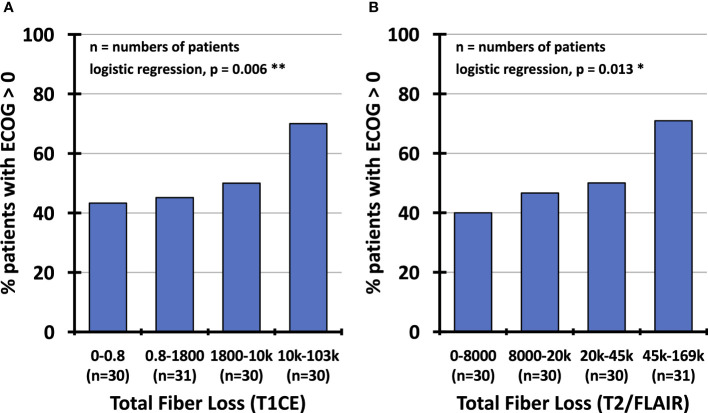
Percentage of patients with impaired performance status (ECOG score≥1) depending on total fiber loss (1 – (patient fiber density/reference fiber density) multiplied by lesion volume), partitioned into quartiles of observed values confined to contrast-enhancing lesions **(A)** and hyperintense T2/FLAIR regions **(B)**. ECOG, Eastern Cooperative Oncology Group; T1CE, T1-weighted contrast-enhancing; T2/FLAIR, T2-weighted fluid-attenuated inversion recovery.

The regression analysis on general performance revealed a significant influence of the total fiber loss in contrast-enhancing lesions (p=0.006) and T2/FLAIR hyperintense areas (p=0.013) on the performance status (ECOG score) of the patients (**Table 3**; **Figure 8**). None of the clinical variables comprising age, gender, type of resection, grade 3 vs. 4, number of surgical procedures, number of radiotherapy series, number of chemotherapy courses and follow-up interval had a significant impact on the ECOG score. In a multivariate logistic regression analysis that included the total fiber loss caused by the 4 different lesion types, only the effect of contrast-enhancing lesions on the ECOG performance status kept its significant impact (p=0.04).

**Table 3:** Results of logistic regression analyses for the impact of total fiber loss on general performance status (normal ECOG=0 vs. impaired ECOG ≥ 1).

Imaging finding^#^	Total fiber loss median (range)	p-value univariate	p-value multivariate
Resection cavity	7506 (0 - 171033)	0.906	0.692
Increased FET uptake	3977 (0 - 204533)	0.054	0.462
Contrast enhancement	1774 (0 - 102088)	0.006**	0.040*
T2/FLAIR hyperintensities	20265 (0 - 168450)	0.013*	0.310

FET, O-(2-[^18^F]fluoroethyl)-L-tyrosine; T2/FLAIR, T2-weighted fluid-attenuated inversion recovery; *p<0.05; **p<0.01; ^#^including patients not affected by respective lesion type (lesion-specific volume/total fiber loss set to zero).

## Discussion

### Main findings

This study shows that structural and metabolic imaging changes after multimodal therapy in glioma patients are associated with a significant reduction in local white matter fiber density, as assessed using the DWI single-shell 3-tissue CSD algorithm. Compared to a matched cohort of healthy subjects, the reduction was almost total in resection cavities, strong in contrast-enhancing lesions and regions with pathologically increased FET PET uptake, and still pronounced in regions with T2/FLAIR hyperintensity. For lesions with an increased FET uptake, an inverse linear relationship between the TBR and a reduced fiber density was observed. The total fiber loss in contrast-enhancing lesions and T2/FLAIR hyperintense regions was associated with a significant risk of lowered performance status as assessed by the ECOG score, while the total fiber loss caused by resection and regions with increased FET uptake did not impact general performance. The methodological issues and clinical implications of these results are discussed below.

### Reliability of CSD-based tractography and fiber density estimation

All tractography methods are based on assumptions that relate the observed non-isotropic diffusion-weighted MRI signal to the expected local fiber architecture. The main prerequisites of these models for reliable performance are a high angular resolution of the diffusion-weighted signal and low confounding of the fiber-associated signal by other sources, e.g., the presence of freely diffusing water (54, 55) or isotropic diffusion such as present in grey matter. The HARDI scheme applied here comprised 120 directions at a high *b*-value of 2700 s/mm^2^, which ensures optimal contrast-to-noise properties within the shell and between the single shell and the *b*=0 data as detailed in (43). From these diffusion-weighted data, the composition of every voxel in terms of grey-matter-like, cerebrospinal fluid-like and white-matter-like tissue was computed and the respective response functions were determined from the experimental data themselves (45). Then, the FODs of the white-matter-like compartments of normal and pathological brain tissue and the contamination of each voxel by freely diffusing water as found in cerebrospinal fluid or edema (48) were calculated by the 3-tissue CSD algorithm (56, 57) for subsequent tractography. Thus, effective measures have been taken to avoid underestimation of fiber density in brain regions affected by tumor, edema, or other pathological tissue (28, 32, 41, 42).

Due to the inhomogeneous distribution of the fiber tracts in the normal brain, reference regions for fiber density have to be carefully selected. This is probably why in a comparable study on fiber density in different tumor compartments of glioblastoma (58) reported in due course after the publication of the method (51, 52), a paradoxical positive correlation between the extent of tumor infiltration and the fiber density was observed. In the present paper, the spatially co-registered data of healthy subjects served as reference, and all procedures were equally applied to the patient and healthy subject data, resulting in reasonable fiber density distributions in both groups. In summary, the methods applied here can be expected to provide a solid estimate of the local and relative fiber density in pathologically altered brain tissue.

### Fiber density in glioma tissue

Several groups investigated the impact of tumor cell density in gliomas’ core or infiltration zone on local fiber density. For this purpose, Stadlbauer et al. (59) applied DTI-based fiber density mapping in patients with non-enhancing WHO grade II or III glioma and evaluated 38 biopsies taken from the tumor center, transition zone, and tumor border. A steep decrease in the fiber density from the periphery into the tumor center was observed. However, the fiber loss in the tumor core was probably overestimated mainly due to the above-mentioned methodological limitations to detect fibers in pathologically altered brain tissue. In a subsequent study, the choline concentration in the tissue (determined by MR spectroscopy), which is a marker of membrane turnover and cellular density, was also inversely related to the fiber density (60). It is worth mentioning that fiber density maps have also been applied to evaluate tumor infiltration of the corticospinal tract in motor-eloquent WHO grade III or IV gliomas, where a significant reduction in fiber density was found in the peritumoral region as well as in the cortico-spinal tract itself (61). The observation that an increase in FET uptake is associated with a decrease in fiber density fits well into these findings, as the FET signal was found to correlate with the tumor cell density in glioma (62, 63). A particular effort was undertaken in the present study to generate reliable reference regions by using spatially registered normal brains and excluding patients with tumor localizations distributed over brain regions with strongly varying physiological fiber density.

### Fiber density in peritumoral edema and gliosis

It is commonly believed that malignant brain tumors disrupt the blood-brain barrier, causing intravascular fluid to leak into the interstitial space and leading to what has been termed as vasogenic edema (64, 65). This process leads to an increased interstitial pressure, which can displace, compress, or disrupt the axons that pass through the affected edematous region (66). In the preoperative setting, several methodological attempts have been made to reliably identify the major fibers tracts in the perilesional tissue of gliomas, including techniques such as free-water modeling (48) and local connectivity mapping (28), which seem to recover more fibers in the tracts than the standard methods based on DTI. We have attempted here for the first time to quantify the relative fiber loss caused by edema and found it to be in the range of 50%.

Late side effects of radiotherapy have been found to mainly affect white matter, leading to demyelination, axonal degeneration, and astrogliosis (15), which, in the absence of other tissue changes, may be readily detected by a signal increase in T2-weighted or FLAIR images (67). In order to quantify radiation-induced white matter damage, surrogate markers for structural connectivity such as cortical atrophy, fractional anisotropy, and mean, axial and radial diffusivity determined from DTI have been applied (22, 68–72). However, in most of these studies, the analysis was performed on the whole brain or confined to anatomically predefined regions or tracts. In contrast, we attempted here to quantify the relative fiber loss in brain regions affected by radiation-induced damage, which was also approximately 50%; the same order of magnitude as the loss caused by edema. However, as the distinction between edema and radiation-induced gliosis proved difficult, these numbers have to be taken as largely provisional.

### Clinical impact of fiber loss

It remains unclear how the apparent loss of fiber density detected by MRI tractography methods is related to neuronal function, e.g., the propagation of action potentials along the axons in the affected tracts. At least in the case of edema, axons are still present but may become nonfunctional, for example, because of compression. However, our data show that the overall extent of fiber loss in the volumes affected by different lesion types significantly affects the global performance status. The differential impact of the lesion types can be explained, at least in part, by the study population. Most patients had recovered from surgery without permanent neurological deficits but were at constant risk of developing radiation-induced damage or recurrent tumor, the latter diagnosed early enough by FET PET before it led to performance loss.

### Limitations of the study

In the present study, regular follow-up data were available for only a few patients. Consequently, each patient was examined only once, resulting in a wide dispersion of intervals between the initiation of therapy and imaging. Because multiple types of lesions were present simultaneously in almost all patients, uncertainties remained regarding the nature and boundaries of each segment, despite all efforts to achieve a distinct and well-circumscribed segmentation. Often, the contrast-enhancing and FET uptaking lesions partially overlapped. However, in a small set of non-overlapping contrast enhancing (n=24) or FET uptake lesions (n=9), a relative fiber density in the same order of magnitude as found before (56% and 54%) was observed. Also, the distinction between edema and radiation-induced gliosis proved difficult, such that a substantial proportion of patients could not be classified. Nevertheless, the relative fiber densities in these two lesion types did not differ significantly. In addition, hyperintense T2/FLAIR lesions could result from tumor infiltration not leading to contrast enhancement or pathologic FET uptake.

The minimal remaining fiber density within the resection cavity can be explained in part by the difficulty of unambiguous segmentation of the resection cavity boundaries. Therefore, it is possible that individual voxels that still contained tissue were part of the segmented area. On the other hand, it should be kept in mind that no model perfectly reflects reality, which in this case obviously resulted in a small number of false positive fibers inside the resection cavity. These problems are illustrated in **Figure 2**.

## Conclusions

In summary, we interpret this study as follows: i) The almost complete fiber loss in the resection cavities was mainly the result of carefully planned neurosurgical interventions based on neuroanatomic and neuro-functional expertise, avoiding neurological deficits in most patients. ii) Most contrast-enhancing lesions were caused by recurrent tumor growth, which severely disrupted fiber tracts in deliberate localizations and thus impacted general performance significantly. iii) Most regions with increased FET uptake also resulted from recurrent tumor growth; however, due to the higher sensitivity of amino acid PET compared to MRI for detecting early tumor infiltration, the associated fiber density loss was less pronounced and did not impair general performance. iv) T2/FLAIR-hyperintense lesions mainly resulted from radiation injury or peritumoral edema or a combination thereof and affected larger brain areas. Although the reduction in fiber density was less pronounced, the larger affected brain volume likely led to dysfunction in many brain regions, resulting in impaired general performance.

## Data availability statement

Data that support the findings of this study are available on reasonable request from the corresponding author. The data are not publicly available due to privacy or ethical restrictions.

## Ethics statement

The study protocol was approved by the ethics committee of the University of Cologne, protocol no. 17-365. Informed written consent according to the Declaration of Helsinki was obtained from all patients and healthy subjects.

## Author contributions

MF and MK analysed and interpreted most of the data. EF, PL, CL, and NJS developed the MR and PET imaging techniques. SC provided the control group data. GS and CPF performed the patient examinations. CWS, MIR, KJL, GRF, NG and MK recruited the patients and conceptualized the study. All authors contributed to the article and approved the submitted version.

## Funding

This project was partially funded by the German National Cohort and the 1000BRAINS-Study of the Institute of Neuroscience and Medicine, Research Centre Juelich, Germany. We thank the Heinz Nixdorf Foundation (Germany) for the generous support of the Heinz Nixdorf Study. We thank the investigative group and the study staff of the Heinz Nixdorf Recall Study and 1000BRAINS. This project has received funding from the European Union’s Horizon 2020 Research and Innovation Programme under Grant Agreement No. 945539 (HBP SGA3; SC) as well as from the Initiative and Networking Fund of the Helmholtz Association (SC). Open access publication is funded by the Deutsche Forschungsgemeinschaft (DFG, German Research Foundation, contract number 491111487).

## Acknowledgments

The technical assistance of N. Judov, S. Frensch, T. Plum, S. Schaden, and L. Tellmann is gratefully acknowledged.

## Conflict of interest

The authors declare that the research was conducted in the absence of any commercial or financial relationships that could be construed as a potential conflict of interest.

## Publisher’s note

All claims expressed in this article are solely those of the authors and do not necessarily represent those of their affiliated organizations, or those of the publisher, the editors and the reviewers. Any product that may be evaluated in this article, or claim that may be made by its manufacturer, is not guaranteed or endorsed by the publisher.
